# Supplementary material for: Synergic Effect of the Antimicrobial Peptide ToAP2 and Fluconazole on Candida albicans Biofilms
Source: Int J Mol Sci. 2024 Jul 16;25(14):7769. doi: 10.3390/ijms25147769 (PMC11276877; doi:10.3390/ijms25147769)
Supplement: Supplementary file 1 [file ijms-25-07769-s001.zip › ijms-3058305-supplementary.pdf]

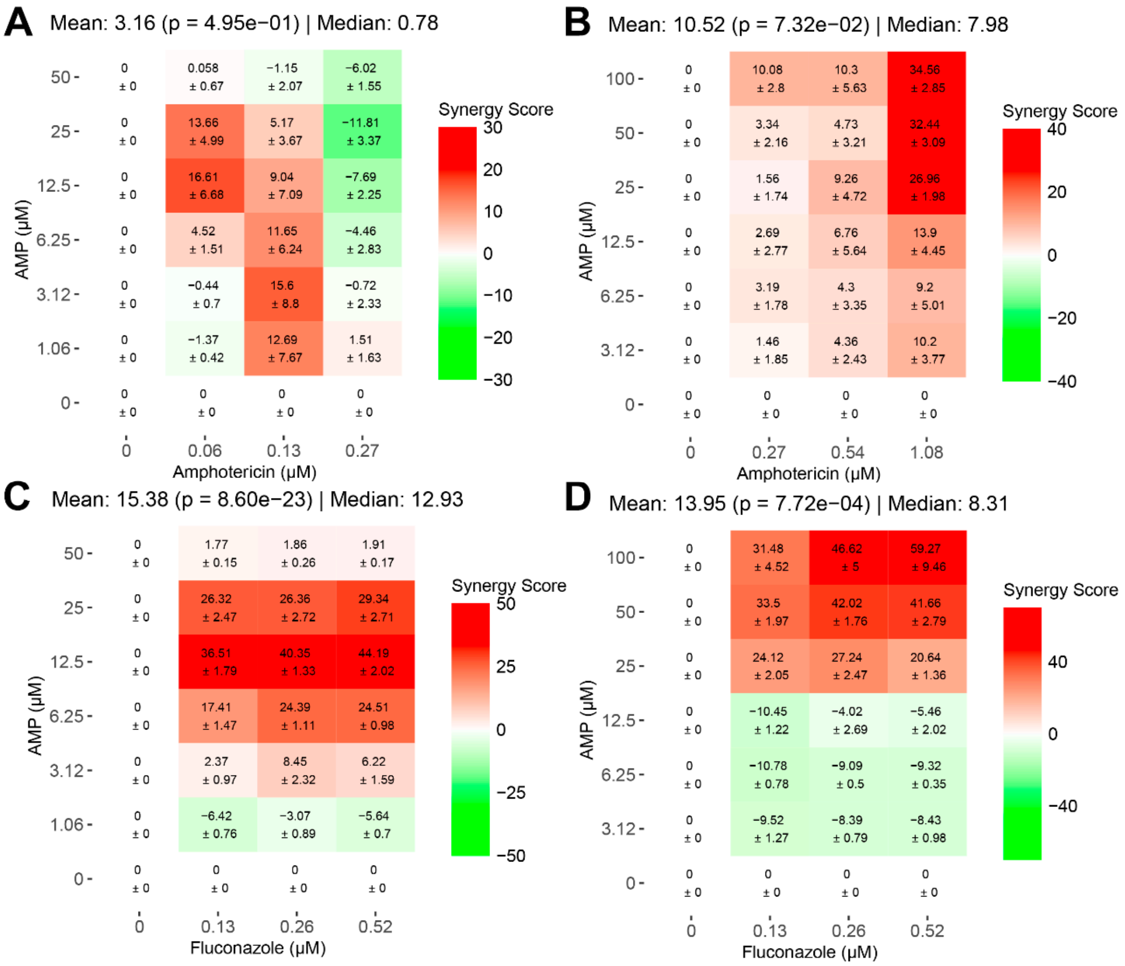

Figure S1. Synergy interaction map in biofilm after treatment with ToAP2 and conventional antifungals. (A and B) Synergy score of early-phase (4h) and mature (24h) *C. albicans* biofilms after 24h treatment with ToAP2 and/or amphotericin B, respectively. (C and D) Synergy score of early-phase (4h) and mature (24h) *C. albicans* biofilms after 24h treatment with ToAP2 and/or fluconazole, respectively. The axes represent the concentrations for ToAP2 (rows) and each antifungal (columns). The heatmap scales vary from red, indicating a synergistic effect, to green, indicating an antagonistic effect. Data are presented as mean ± standard error of the mean of three independent assays.
